# How does the turnover intention of village clinic doctors change after China's new health care system reform? A comparison based on three surveys in a province in eastern China

**DOI:** 10.3389/fpubh.2022.1092386

**Published:** 2023-01-05

**Authors:** Haiyuan Lv, Lifang Zhou, Zhaofeng Yu, Jiaxian Shao, Yuncong Yu, Wenqiang Yin, Zhongming Chen

**Affiliations:** ^1^School of Management, Weifang Medical University, Weifang, Shandong, China; ^2^Human Resources Office, Weifang Medical University, Weifang, Shandong, China

**Keywords:** new health care system reform, village clinic doctors, turnover intention, human resources for health, occupational psychology

## Abstract

**Objectives:**

This study aims to analyze the changes in village clinic doctors' turnover intention following the new health care system reform (NHCSR).

**Methods:**

All the data were obtained from three surveys conducted in 2012, 2015, and 2018 in a province in eastern China. Descriptive analysis and analysis of variance (ANOVA) were used to analyze the changes in village clinic doctors' turnover intention.

**Results:**

The mean scores of village clinic doctors' turnover intention were 2.87 ± 1.07, 2.76 ± 1.12, and 2.99 ± 1.14 in 2012, 2015, and 2018, respectively, with a significant difference (*F* = 3.60, *P* = 0.03). During the 3 years, the turnover intention scores of village clinic doctors who were male, aged 40 years and below, secondary technical school graduates, had the qualifications to practice as village clinic doctors, and were under village-township integrated management first decreased and then increased. The turnover intention scores of village clinic doctors with medical practitioner qualifications increased; however, the scores, decreased for those without village-township integrated management.

**Conclusion:**

The turnover intention of village clinic doctors has increased, and the possible reasons for this phenomenon include changes in the demographic characteristics of village clinic doctors and policy factors. The government should pay more attention to the problems that village clinic doctors consider of urgent concern, such as the treatment of income and pension insurance, and provide greater career development and training opportunities for village clinic doctors to maintain their motivation and stability.

## Introduction

Due to an increase in the aging population, people's health needs are increasing (1). The imbalance between the supply of human resources for health and public needs has become increasingly evident, particularly in rural areas. Previous studies (2–4) have shown that the shortage of human resources for health in rural areas is a common problem worldwide. In China, village clinic doctors provide primary health services in rural areas (5). Their main responsibilities are to provide primary medical and public health services for rural residents, such as diagnosing and treating common diseases, health management of hypertension and diabetes, health management for elderly individuals, and the management of resident health records (6–8). They serve as the backbone of health services in rural areas (9) and protect the health of rural residents (10), thereby forming the basis of China's county–township–village three-tiered health service system network. In 2021, the number of village clinic doctors in China was 671,298, 81.5% of whom had secondary school and college degrees, while only 6.7% had bachelor's degree or above. A total of 34% of village clinic doctors were between 45 and 54 years of age, and 28.8% were 60 and older (11). The quantity and quality of village clinic doctors are closely related to the performance of the overall National Health Service system and the health levels of rural residents (12).

Turnover intention is a conscious and deliberate willfulness to leave an organization (13). Previous studies have shown that high turnover intention means high actual turnover behavior (14–18). Village clinic doctors are an indispensable part of health service provision in rural areas. Turnover intention among village clinic doctors is directly related to the stability of the three-tiered health service system network and the accessibility of health services for rural residents (7).

In 2009, China launched the new health care system reform (NHCSR), which adheres to the basic principles of “ensuring the basic health needs of residents, strengthening the primary health care system, and establishing a health operation support system” (19). The Chinese government strengthened the construction of the primary health service system which includes village clinic doctors, by implementing the essential medicine system, providing primary public health services, and improving the basic medical insurance system (14). In order to regulate the prescription behavior of village clinic doctors, the NHCSR requires that village clinic doctors only use essential medicine in medical services, and states that they must adhere to zero mark-up sales. They are also prohibited from using non-essential drugs and making profits from drugs (20). Furthermore, the NHCSR asked village clinic doctors to provide health management services for patients suffering from hypertension and diabetes, for the purpose of improving the health status of patients with chronic diseases. To improve the capabilities of village clinic doctors, better training is important, which will help to improve health care in rural areas (21). With the implementation of the NHCSR, the management mechanism for village clinic doctors has been gradually improved (22), and the government's management standards for qualifications, drug use, and the operation of village clinics have also become more stringent (23). This reform is beneficial to the promotion of population health and the development of health service in the country. However, it has placed increased pressure on village clinic doctors without improving their job rewards (24). Therefore, it has had a negative effect on the job stability of village clinic doctors.

Since the NHCSR was implemented, health management researchers have conducted numerous studies on the turnover intention of village clinic doctors. Sun (25) described the current situation of the turnover intention of village clinic doctors in Wuhu, Anhui Province, and the results showed that the mean score of village clinic doctors' turnover intention was high, with a value of 10.61 ± 4.43. According to the study, an important factor behind this phenomenon is that the implementation of the NHCSR has greatly reduced the income of village clinic doctors, which makes it impossible to maintain the normal life of village clinic doctors; this has resulted in a high turnover intention. Xie et al. (26) studied village clinic doctors' turnover intention in Huailai County, Hebei Province, and the results showed that their turnover intention was high, with a value of 2.67 ± 0.22. They found that the NHCSR resulted in high workloads and low wages for village clinic doctors, leading to dissatisfaction and a high turnover intention. A survey of village clinic doctors from Hubei Province was conducted by Fang et al. (6) showed that 36.8% of village clinic doctors were considering leaving their current positions. They found that the implementation of the NHCSR failed to solve the problems of pension insurance and the insufficient training of village clinic doctors, which reduced their job satisfaction and strengthened their turnover intention.

Previous studies have shown that village clinic doctors have high turnover intention. The main factors affecting turnover intention among village clinic doctors are salary, work engagement and output, workload, working conditions, identity issues, pension insurance, incentive mechanisms, occupation mentality, development prospects and so on (27–32). Although previous studies provide a good reference for this study, most are cross-sectional studies conducted at a particular moment in time. Therefore, the number of studies that reflect the overall changes in the turnover intention of village clinic doctors since the implementation of the NHCSR is small. Based on the data of the three surveys on village clinic doctors conducted in a province in eastern China in 2012, 2015, and 2018, our study analyzes the changes in the turnover intention of village clinic doctors since the implementation of the NHCSR and tries to explain the possible reasons behind these changes from the perspective of the NHCSR. This study reflects the impact of the NHCSR on the turnover intention of village clinic doctors. It suggests that researchers and health administrators should not only pay attention to the regulation of resources and the optimization of health services, but also the occupational mentality of human resources for health in rural areas to ensure the stability of village clinic doctors during the implementation of the NHCSR. This study can provide a reference for improving the stability of village clinic doctors.

## Materials and methods

### Study design and sampling

The data analyzed in this study were obtained from three surveys of village clinic doctors practicing in a province in eastern China in 2012, 2015, and 2018, with 408, 519, and 223 valid questionnaires and response rates of 93.6, 94.3, and 92.9%, respectively (5). In 2012, we conducted a baseline survey using a multistage random sampling method. First, according to the level of economic development, three prefecture-level cities were selected from 17 prefecture-level cities in the province that represented economically developed areas, moderately developed areas and underdeveloped areas. Then, three counties with different economic development levels were randomly selected from each prefecture-level city, three townships were randomly selected from each county, and one village clinic doctor was randomly selected from each village clinic in the three townships to fill in the questionnaire in the township health center. In 2015 and 2018, follow-up surveys were conducted with the same sampling method in these township health centers. In 2015, the provincial government implemented the policy of “One village clinic per village.” Therefore, the number of village clinics in every township increased rapidly, leading to the sample size of the second survey being larger than in 2012. However, in 2018, due to urbanization and regional health development, some village clinics were closed or merged with others and could not be located. In addition, the demission and retirement of village clinic doctors (6) led to a reduction in the sample size in 2018.

### Measurement instruments

The questionnaire includes demographic variables and the turnover intention of village clinic doctors. Turnover intention was measured according to the method of Mobley et al. (33) and Farh et al. (34). The scale consists of three items, namely, “I want to leave my present position,” “I don't want to be a village clinic doctor” and “I want to find a new job.” All the items were rated on a five-point Likert scale that ranged from 1 to 5, wherein 1 represents “very inconsistent,” 2 represents “relatively inconsistent,” 3 represents “inconclusive,” 4 represents “relatively consistent,” and 5 represents “very consistent.” The higher the score was, the stronger the turnover intention. The overall score of the turnover intention was the average score of three items. Cronbach's alpha coefficient of this study scale was 0.834, with good reliability. In terms of the validity test, the KMO value of the scale was 0.69, and the χ^2^ of Bartlett's test of sphericity was 476.83 (df = 3), indicating that it was suitable for factor analysis. Exploratory factor analysis (EFA) and varimax rotation were used to test the construct validity of the questionnaire. As a result, one common factor was extracted from the three observed items, and the cumulative variance contribution rate was 75%. The factor loadings of items were between 0.843 and 0.91, indicating that the questionnaire had good internal structural validity.

### Statistical analysis

First, a descriptive analysis was conducted on the demographic characteristics and the turnover intention of village clinic doctors. Then, a *t-test*, an LSD test, and one-way ANOVA were used to compare the differences between groups and years. The significance level of all tests was set at *P* < 0.05 (two-tailed).

## Results

### Demographic characteristics

The demographic characteristics of the village clinic doctors in the three surveys in 2012, 2015, and 2018 are shown in Table 1. The proportion of males was above 60% in all three surveys. In terms of educational background, more than 90% of the village clinic doctors had received a secondary school education or higher in all three survey years. In terms of age distribution, in 2012 and 2015, most village clinic doctors were aged 40 years and below. The number of village doctors aged 41–50 was the largest, reaching 51.36% in 2018, indicating that the aging of village clinic doctors is an increasingly prominent issue. In all three surveys, 58% or more of the village clinic doctors held qualifications to practice as village clinic doctor. Meanwhile, most of the village clinics where the surveyed doctors practice are involved in the village-township integrated management of village health services in local township health centers.

**Table 1:** Demographic characteristics of village clinic doctors from three surveys.

**Characteristics**	**2012 (*N* = 408)**	**2015 (*N* = 519)**	**2018 (*N* = 223)**
	***N* (%)**	***N* (%)**	***N* (%)**
**Gender**			
Male	245 (60.49)	342 (65.90)	158 (71.17)
Female	160 (39.51)	177 (34.10)	64 (28.83)
**Age**			
≤40	202 (50.63)	235 (45.28)	54 (24.55)
41~	94 (23.56)	171 (32.95)	113 (51.36)
51~	103 (25.81)	113 (21.77)	53 (24.09)
**Educational background**			
No medical educational background	35 (8.66)	39 (7.60)	8 (3.60)
Secondary technical school graduates	305 (75.50)	386 (75.24)	160 (72.07)
Junior college degree or higher	64 (15.84)	88 (17.15)	54 (24.32)
**Qualification**			
Medical practitioner	112 (27.72)	34 (6.55)	22 (9.87)
Assistant medical practitioner	50 (12.38)	33 (6.36)	40 (17.94)
Qualification to practice as village clinic doctors	242 (59.90)	452 (87.09)	161 (72.20)
**Village-township integrated management**			
Yes	344 (87.76)	495 (96.12)	218 (97.76)
No	48 (12.24)	20 (3.88)	5 (2.24)

Percentage of village clinic doctors with missing data for gender (0.7% in 2012, 0.4% in 2018), age (2.2% in 2012, 1.3% in 2018), educational background (1.0% in 2012, 1.2% in 2015, 0.4% in 2018), qualification (1.0% in 2012), and village-township integrated management (3.9% in 2012, 0.8% in 2015, 0.4% in 2018).

### Turnover intention total score comparison

The overall average scores of the turnover intention among the village clinic doctors in the three surveys are shown in Table 2. In 2012, 2015, and 2018, the mean scores of turnover intention of village clinic doctors were 2.87 ± 1.07, 2.76 ± 1.12, and 2.99 ± 1.14, respectively, showing an upward trend (*F* = 3.60, *P* = 0.03). The results of the LSD showed that there was no statistical difference between 2012 and the other 2 years. However, the score in 2018 was significantly higher than that in 2015.

**Table 2:** Comparison of village clinic doctors' turnover intention scores from three surveys.

**Characteristics**	**2012**	**2015**	**2018**	***F***	***P***
**Gender**					
Male	2.86 ± 1.08	2.75 ± 1.14	3.03 ± 1.13	3.33	0.04
Female	2.91 ± 1.06	2.77 ± 1.09	2.87 ± 1.15	0.67	0.51
*t*	−0.46	−0.24	0.92		
*P*	0.64	0.81	0.36		
**Age**					
≤40	3.05 ± 1.02	2.75 ± 1.08	3.01 ± 1.13	4.78	<0.01
41~	2.80 ± 1.09	2.86 ± 1.17	2.98 ± 1.13	0.72	0.49
51~	2.54 ± 1.08	2.62 ± 1.13	2.96 ± 1.22	2.48	0.09
*F*	7.74	1.51	0.03		
*P*	<0.01	0.22	0.98		
**Educational background**					
No medical educational background	2.55 ± 1.02	2.57 ± 1.05	2.92 ± 1.46	0.39	0.68
Secondary technical school graduates	2.92 ± 1.09	2.78 ± 1.13	3.04 ± 1.13	3.25	0.04
Junior college degree or higher	2.84 ± 0.98	2.67 ± 1.13	2.86 ± 1.12	0.68	0.51
*F*	1.61	0.84	0.48		
*P*	0.2	0.43	0.62		
**Qualification**					
Medical practitioner	2.56 ± 1.11	2.64 ± 0.87	3.23 ± 1.19	3.47	0.03
Assistant medical practitioner	2.81 ± 1.10	2.78 ± 1.05	3.11 ± 1.18	1.05	0.35
Qualification to practice as village clinic doctors	3.02 ± 1.01	2.76 ± 1.15	2.92 ± 1.12	4.35	0.01
*F*	6.79	0.21	0.98		
*P*	<0.01	0.82	0.38		
**Village-township integrated management**					
Yes	2.84 ± 1.06	2.74 ± 1.13	3.02 ± 1.13	4.48	0.01
No	3.12 ± 1.08	2.85 ± 1.07	1.73 ± 0.43	4.05	0.02
*t*	−1.65	−0.41	2.52		
*P*	0.1	0.68	0.01		
**Total turnover intention**					
Mean total score	2.87 ± 1.07	2.76 ± 1.12	2.99 ± 1.14	3.6	0.03

### Turnover intention scores of village clinic doctors with different characteristics

The turnover intention scores of village clinic doctors with different characteristics are shown in Table 2. In 2012, village clinic doctors of different ages had different turnover intention scores. Younger village clinic doctors had higher turnover intention scores (*F* = 7.74, *P* < 0.01). Specifically, the score of village clinic doctors aged 40 years and below was the highest, with a value of 3.05 ± 1.02, while that of village doctors aged 51 years and above was the lowest, with a value of 2.54 ± 1.08. There were significant differences in the turnover intention of village clinic doctors with different qualifications (*F* = 6. 79, *P* = 0.01). Village clinic doctors with the qualifications to practice as village clinic doctors had the highest score, of 3.02 ± 1.01, while those with the qualifications of medical practitioners had the lowest score, of 2.56 ± 1.11. In 2018, village-township integrated management had a significant impact on the turnover intention of village clinic doctors (*t* = 2.52, *P* = 0.01), and the turnover intention score of village clinic doctors who had implemented village-township integrated management was 3.02 ± 1.13, which was higher than that of others. In 2015, there were no significant differences in turnover intention among village clinic doctors with different characteristics.

### Changes in the turnover intention scores of village clinic doctors with different characteristics over the 3 years

Changes in the turnover intention of village clinic doctors with different characteristics over the 3 years are shown in Table 2. In terms of gender, the scores of male village clinic doctors' turnover intention decreased at first and then increased (*F* = 3.33, *P* = 0.04), with values of 2.86 ± 1.08, 2.75 ± 1.14, and 3.03 ± 1.13, respectively. In terms of age, the turnover intention scores of village clinic doctors aged 40 years and below decreased at first and then increased in the three years (*F* = 3.78, *P* < 0.01), with values of 3.05 ± 1.02, 2.75 ± 1.08, and 3.01 ± 1.13, respectively. In terms of educational background, the turnover intention scores of village clinic doctors with a technical secondary school education first decreased and then increased (*F* = 3.25, *P* = 0.04), with values of 2.92 ± 1.09, 2.78 ± 1.13, and 3.04 ± 1.13, respectively. In terms of qualifications to practice, the turnover intention scores of village clinic doctors with the qualifications of medical practitioners increased (*F* = 3.47, *P* = 0.03), with values of 2.56 ± 1.11, 2.64 ± 0.87, and 3.23 ± 1.19, respectively, and the scores of village clinic doctors with the qualifications of village clinic doctors first decreased and then increased (*F* = 4.35, *P* = 0.01), with values of 3.02 ± 1.01, 2.76 ± 1.15, and 2.92 ± 1.12, respectively. In terms of village-township integrated management, the turnover intention scores of village clinic doctors under village-township integrated management first decreased and then increased (*F* = 4.48, *P* = 0.01), with values of 2.84 ± 1.06, 2.74 ± 1.13, and 3.02 ± 1.13, respectively. The turnover intention scores of village clinic doctors without village-township integrated management decreased (*F* = 4.05, *P* = 0.02), with values of 3.12 ± 1.08, 2.85 ± 1.07, and 1.73 ± 0.43, respectively.

## Discussion

In the implementation process of the NHCSR, the turnover intention of village clinic doctors showed an upward trend, and the overall level was high, which is consistent with the studies of Sun et al. (35) and Li (36). According to the data analysis, although there was no significant difference in the scores of higher turnover intentions between 2012 and 2015, there was a significant increase in 2018. The possible reasons for this phenomenon include demographic changes among characteristics of village clinic doctors and policy factors.

In terms of demographic characteristics, first, the proportion of male village clinic doctors increased, and their turnover intention scores increased, which led to an increase in the overall turnover intention score. The possible reason for this phenomenon is that in rural society, compared with female village clinic doctors, male village clinic doctors have been found to bear greater work pressure and social responsibility, meaning they may pay more attention to the efforts and rewards of work. Previous studies have shown that the imbalance between the efforts and rewards of village clinic doctors' work has led to the rise of the turnover intention of male village clinic doctors (12, 37). Second, the proportion of elderly village clinic doctors increased, and their scores increased. The pension insurance level was low (38), and the approaching retirement of elderly village clinic doctors was the reason for their high turnover intention (18). Third, in the implementation process of the NHCSR, the proportion of village clinics participating in village-township integrated management increased; therefore, village clinic doctors' turnover intention scores increased. After the implementation of village-township integrated management, township hospitals had unified management of the finances, personnel, medicines, and services of village clinics. This has fundamentally changed the way that village clinic doctors practice, and their autonomy at work has been limited to some extent (39, 40). Simultaneously, under this management pattern, the “siphon effect” of township hospitals on village clinics intensified, and the original economic interests of village clinic doctors were taken over by township hospitals (14, 39, 41–43), which damaged the interests of village clinic doctors.

In terms of policy, village clinic doctors are facing serious challenges brought by the NHCSR. First, there was an imbalance between the effort and reward of village clinic doctors. Before the implementation of the NHCSR, village clinic doctors provided only primary medical services to residents in rural areas. After the implementation of the NHCSR, village clinic doctors were required to provide a large number of public health services (44, 45), especially after 2018, when the content of essential public health services increased, and the performance appraisal became increasingly stringent, which led to a heavier workload and an imbalance between work engagement and actual return (46, 47). Second, in the implementation process of the NHCSR, the income level of village clinic doctors decreased. Previous studies showed that before the implementation of the NHCSR, the average annual income of village doctors was 22,543 yuan. After the implementation of the NHCSR, the average annual income of village clinic doctors dropped to 13,624.9 yuan (35). For a long time, the personal incomes of village clinic doctors depended on drug profits greatly (48). In the implementation process of the NHCSR, essential drugs were sold at a zero-mark-up price (49), which means that village clinic doctors cannot benefit from drug profits. In ~2016, the policy achieved full coverage in the province, so the turnover intention of village clinic doctors increased significantly in 2018. However, government subsidies cannot compensate for the loss of village clinic doctors' incomes caused by the essential medicine system, resulting in the lack of income security for village clinic doctors. Third, the security mechanism for village clinic doctors' pensions insurance is not perfect (50). Mosadeghrad et al. (51) argued that a lack of wages and a perfect incentive mechanism are increasing sources of frustration and contribute to employee turnover. Employees can work effectively only if their essential needs have been met. Limited to the status of being both farmers and doctors, they cannot enjoy the government's wages and benefits, and the level of income and security is also on the low side (52, 53). Fourth, in the implementation process of the NHCSR, village clinic doctors have had to undertake a higher workload. This has negatively affected service quality and doctor-patient communication. Workplace violence caused by doctor–patient disputes often occur in China (54–57), which leads to great psychological pressure on village clinic doctors. Fifth, with the implementation of the NHCSR, the social status of village clinic doctors has greatly declined (58). Compared with rural teachers who also work in rural areas (45), there is a large gap between these two groups in terms of pension insurance, treatment of income, medical insurance and supportive policies, leading village clinic doctors to feel unfairly treated, resulting in “relative deprivation” and turnover intention (59).

### Strengths and limitations

This is the first long-term study to monitor the turnover intention of village clinic doctors in the implementation process of the NHCSR in China. In this study, a *t*-test, an LSD test, and one-way ANOVA were used to explore the influencing factors of turnover intention of village clinic doctors. However, due to the limitation of data, the study does not use regression analyses to analyze the influencing factors of village clinic doctors' turnover intention under the condition of fully controlling the confounding factors. Most previous studies were cross-sectional studies conducted at a certain moment in time. This study compares three surveys of village clinic doctors and analyzes the changes in village clinic doctors' turnover intention in the implementation process of the NHCSR. This is the strength of this study. However, there are some limitations in this study. First, a self-report questionnaire was used to collect information. The social expectation effect caused by observation bias was inevitable. Second, because influencing factors were not investigated, the reasons for the changes in village clinic doctors' turnover intention may not be limited to those described in this paper. Moreover, our discussion was mainly focused on the impact of the NHCSR on turnover intention of village clinic doctors without illustrating other factors. Third, because the survey sites of village clinic doctors were scattered and there were only 1 or 2 doctors in most village clinics, it was difficult to conduct a large-sample follow-up survey. Fourth, in the survey data from 2018, the sample size of village clinic doctors who had “no medical educational background” and “no village-township integrated management” was too small and may have had a confounding effect on the comparative analysis of the turnover intention of village clinic doctors with different characteristics. Fifth, this study is a repetitive cross-sectional survey, not a cohort study, the instability of the study sample may affect the reliability and generalizability of the results. Affected by the sample size, the results of this study may offer limited representation of the turnover intention of village clinic doctors in China.

## Conclusion

The results of our study show that the score of turnover intention score of village clinic doctors increased during the implementing the NHCSR. The main reasons for this phenomenon are low income, insufficient rewards, inadequate pension insurance, frequent doctor–patient disputes and the growing sense of relative deprivation. Village clinic doctors are an important part of the implementation of the hierarchical medical system. Under the comprehensive action of many binding policies, village clinic doctors are facing enormous challenges in their work. The government should pay attention to treatment of income, pension insurance, doctor–patient dispute protection and other issues experienced by village clinic doctors and standardize career development and job training. Such measures will stabilize the number of village clinic doctors, improve their service capabilities, and provide strong support for the implementation of the hierarchical medical system.

This study has enriched the research on primary care doctors' turnover intention in the implementation process of the NHCSR in China. It explores new research ideas about primary care doctors' turnover intention and provides a useful reference for the follow-up research in terms of research design and ideas. However, in the critical stage of the NHCSR, the career development of village clinic doctors still faces enormous challenges. In the follow-up study, more in-depth qualitative interviews and research should be carried out to support and enhance the results of quantitative research. In recent years, the Chinese government has implemented a series of new measures, such as the construction of an integrated health service system, the training of targeted admission medical education program in rural areas, and the reform of medical insurance payment methods, aiming to strengthen the construction of the health service force in rural areas. Therefore, more systematic and detailed research should be carried out to accurately observe the impact of the NHCSR on the turnover intention of village clinic doctors.

## Data availability statement

The original contributions presented in the study are included in the article/supplementary material, further inquiries can be directed to the corresponding authors.

## Author contributions

HL, LZ, WY, and ZC conceived the study. ZC participated in the acquisition of data. HL, LZ, ZY, JS, and YY analyzed the data. HL and ZY drafted and revised the paper. LZ, ZY, JS, YY, WY, and ZC reviewed the paper. All authors have read and approved the final version of the manuscript.
